# Supplementary material for: Interacting active surfaces: A model for three-dimensional cell aggregates
Source: PLoS Comput Biol. 2022 Dec 16;18(12):e1010762. doi: 10.1371/journal.pcbi.1010762 (PMC9803321; doi:10.1371/journal.pcbi.1010762)
Supplement: S1 Fig — (A) Side view, cut by a plane perpendicular to the adhesion patch of an adhering doublet for D˜ larger than the critical value. The system develops a buckling instability that grows with time. The instability eventually leads to self-intersections (right-most image, where the blue cell has collapsed). (B) Coloured lines: pressure for simulations with different values of l˜ and κ˜. Black dotted line: theoretical approximation valid in the limit of κ˜→0,l˜→0,r˜min→0. (C) Convergence of the method evaluated by computing the inner cell pressure P for different average mesh sizes h, and comparing the results with a simulation with h/ℓ ≈ 2 ⋅ 10−2 (finer). For each h, we compute a box plot using different values of D˜ and fixed κ˜=10-2, l˜=0.02. (D) Side view, cut by a plane perpendicular to the adhesion patch of an adhering doublet with asymmetric tension for α = 0.7; the cell with lower tension (blue) engulfing the cell with higher tension (red) develops a self-intersection in our numerical simulations (right-most image). (PDF) [file pcbi.1010762.s002.pdf]

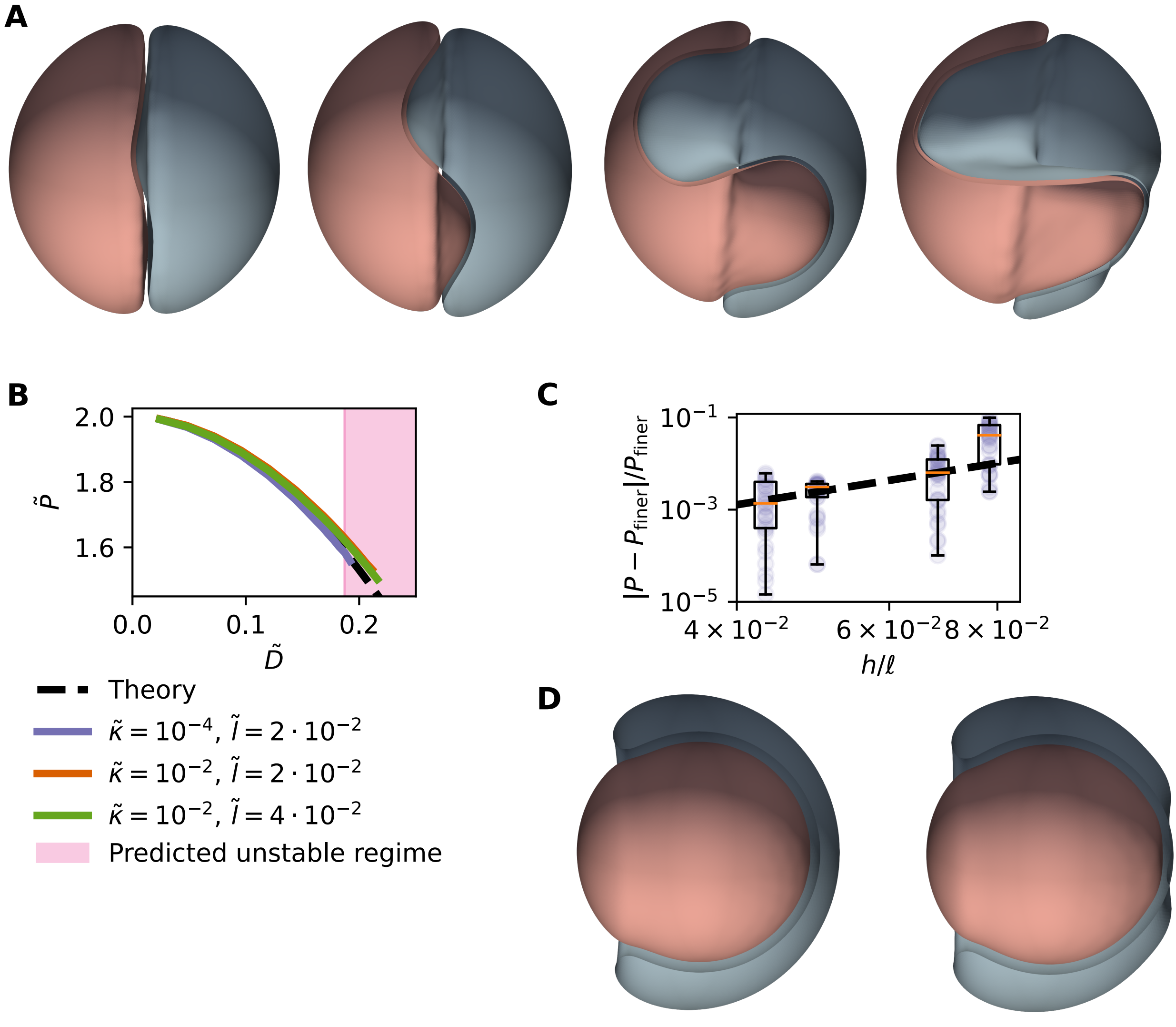

A
B
C
D
2.0
1.8
1.6
$\tilde{P}$
0.0
0.1
0.2
$\tilde{D}$
Theory
$\tilde{\kappa} = 10^{-4}$, $\tilde{l} = 2 \cdot 10^{-2}$
$\tilde{\kappa} = 10^{-2}$, $\tilde{l} = 2 \cdot 10^{-2}$
$\tilde{\kappa} = 10^{-2}$, $\tilde{l} = 4 \cdot 10^{-2}$
Predicted unstable regime
10^{-1}
10^{-3}
10^{-5}
$|P - P_{\mathrm{finer}}|/P_{\mathrm{finer}}$
4×10^{-2}
6×10^{-2}
8×10^{-2}
h/ℓ
