# Supplementary material for: Interacting active surfaces: A model for three-dimensional cell aggregates
Source: PLoS Comput Biol. 2022 Dec 16;18(12):e1010762. doi: 10.1371/journal.pcbi.1010762 (PMC9803321; doi:10.1371/journal.pcbi.1010762)
Supplement: Attachment — Submitted filename: review.pdf [file pcbi.1010762.s006.pdf]

Summary

The study by Torres-Sánchez et al. describes a numerical method to simulate the mechanics of a collection of closed cells. The boundary of each cell is described as a two-dimensional surface, which represents the cell membrane and its attached acto-myosin cortex. The continuum equations governing the evolution of such a two-dimensional surface are taken from prior investigations by the authors (Ref. [52]) and are discretized with a Lagrangian finite element scheme that uses Loop subdivision surfaces to maintain the necessary surface continuity requirements. The numerical method captures in-plane flows and out-of-plane elasticity of each cell, as well as short-range intercellular adhesive forces between cells. The code for this method, written in `C++`, has already been openly released.

Given the variety of physical phenomena incoroporated into this numerical framework, and the public release of the numerical implementation, I believe this manuscript and its accompanying code are of timely interest to scientists in the field of cellular mechanics. Thus, I favor publication in PLOS Computational Biology if the authors can provide a satisfactory response to my three major concerns. One is related to the absence of a mass balance in the governing equations, which I fear may render the numerical implementation invalid for the types of phenomena the authors seek to describe. My second concern is regarding the possibility of lubrication layers to develop in the thin regions between cells. Finally, I would like to see numerical benchmarking for the remeshing procedure.

Major Comments

1. Mass balance and surface tension

I have a major concern regarding the "active tension" contribution to the in-plane stresses, expressed as $\hat{t}_{\mathrm{a}}^{ij} = \gamma g^{ij}$. When γ is prescribed to be a uniform constant, $\nabla_i \hat{t}_{\mathrm{a}}^{ij} = \gamma \nabla_i g^{ij} = 0$ and your active tension does not drive in-plane flows (it only contributes to an effective Laplace pressure in the out-of-plane equation). If γ is prescribed and non-uniform, you provide another forcing term $f_{\mathrm{a}}^i = \nabla^i \gamma$ into the in-plane equations, which drives a flow of material. In both cases, however, you do not include a mass balance in your dynamical description—and so there is no coupling between areal dilations/contractions and the dynamics of the material. I find this extremely problematic: it may render all of your numerical results invalid, as I detail below.

Let us first consider the case where there are no active tensions. In this case, one could model the energetics of area changes as

$$W_{\mathrm{compress}} = \int_{\mathcal{S}} \mathrm{d}S \left\{ \frac{k_{\mathrm{c}}}{J} \left(J - 1 \right)^2 \right\} , \tag{1}$$

where k_{c} is a compression modulus and $J = \rho_0 / \rho$ is the Jacobian determinant of the transformation—see, for example, Eqs. (153), (154), and (159) of *Sahu et al.* Phys. Rev. E **96** (2017). In this case, the contribution to the in-plane stresses is given by

$$\hat{t}_{\mathrm{c}}^{ij} = 2 k_{\mathrm{c}} \left(J - 1 \right) g^{ij} , \tag{2}$$

which leads to an equation of state for the surface tension—written as

$$\gamma_{\mathrm{c}}(\rho) = 2 k_{\mathrm{c}} \left(J - 1 \right) . \tag{3}$$

The resultant in-plane force in the linear momentum balance is given by

$$\nabla_i \hat{t}_{\mathrm{c}}^{ij} = -\frac{2k_{\mathrm{c}} J}{\rho} \nabla^j \rho \ . \tag{4}$$

Such a term is missing from your analysis, and is likely to be important in many of the phenomena you seek to describe. In cases where you prescribe γ to be constant, the surface tension gradients in Eq. (4) would drive a lipid flow that your numerical method currently does not capture. On the other hand, if you prescribe γ to be spatially varying, then surface tension gradients could in fact *balance* the prescribed $\nabla^i \gamma$, and your system could perhaps reach a steady state in which lipids are stationary—though in your current implementation, there must always be an in-plane flow of lipids, even at long times. Without a mass balance in your dynamical description of the system, it is hard to know if your numerical results even qualitatively describe biological situations, and thus your numerical method is incomplete.

I have one additional comment regarding your active tension term. If we think of γ as an effective energy per area, then to me it makes physical sense to think of γ as the product of an energy per mass ξ and the mass per area ρ. Then a "constant" active tension would be expressed as $\xi = \text{const.}$, such that the active stresses would vary as material is compressed in-plane. Indeed, your own earlier work (Ref. [38], §3.3, §5.3) argues that the active surface tension should be expressed as

$$\gamma = \rho \xi(\rho) \ , \tag{5}$$

and then proposes forms for $\xi(\rho)$. Even if ξ were independent of ρ, there is still some effect of active forces on the in-plane flow of materials.

If you insist on prescribing γ with no mass balance, I fear that your numerical method would fail to reproduce the well-known instabilities in cylindrical membranes that have been observed in many experiments, including in live neurons with an intact acto-myosin cortex:

- *Pullarkat et al.* Phys. Rev. Lett. **96** (2006).
- *Datar et al.* Biophys. J. **117** (2019).

In such systems, a global disturbance to the system leads to pearling, which is theoretically known to arise when $\gamma > 3\kappa/(2R^2)$—see, for example, *Narsimhan et al.* J. Fluid Mech. **777** (2015), which includes the effects of both membrane and fluid viscosity in the Appendix. A local perturbation, on the other hand, leads to outwardly propagating fronts, as discussed in *Tchoufag et al.* Phys. Rev. Lett. **128** (2022). I believe your current numerical implementation cannot describe a cylindrical cell which, when subject to different infinitesimal perturbations, evolves in these two different ways.

2. Lubrication layers between cells

To account for the effects of the internal and external medium surrounding the cell, you apply a body force $\boldsymbol{f} = P\boldsymbol{n} - \xi \boldsymbol{v}$ to the cell surface. Here, P is meant to represent the pressure difference across the surface, and is approximated by the Lagrange multiplier enforcing the global volume constraint in Eq. (14). In this case, the internal pressure is assumed constant, and is measured relative to the external pressure—a fixed, reference value. Moreover, if the surface is surrounded by fluid, then the parameter ξ captures the drag due to shear forces, for which

$$\xi \sim \frac{\mu_{\mathrm{f}}}{\ell} \ , \tag{6}$$

where μ_f is the fluid shear viscosity and ℓ is a characteristic length scale (the effective radius you define, for example).

While these approximations seem reasonable for a single cell, I worry about the lubrication effects that arise within the thin layer of fluid separating two cells, especially given that one of the key advancements of your method is the ability to simulate many adjacent cells. Let us consider two cells separated by a thin fluid region of width r_min and length ℓ, with $r_\mathrm{min} \ll \ell$. If we denote the characteristic speed of fluid normal to the cell surface as v_c and that parallel to the surface as u_c, then it is well-known from lubrication theory that

$$\frac{u_\mathrm{c}}{\ell} = \frac{v_\mathrm{c}}{r_\mathrm{min}} \qquad \text{and} \qquad \Delta P_\mathrm{f} = \frac{\mu_\mathrm{f} u_\mathrm{c} \ell}{r_\mathrm{min}^2} \,, \tag{7}$$

where ΔP_f is the pressure drop of the fluid along the length ℓ of thin region [*L. Gary Leal.* Advanced Transport Phenomena, Chapter 5. Cambridge University Press (2007)]. Lubrication pressures can be large, and could dominate the internal pressure P you determine from your volume constraint—yet this effect is missing from your implementation. Moreover, the shear force on the cell by the lubrication fluid now scales as $\mu_\mathrm{f} u_\mathrm{c}/r_\mathrm{min}$, for which the corresponding drag coefficient would be

$$\xi \sim \frac{\mu_\mathrm{f}}{r_\mathrm{min}} \,, \tag{8}$$

which is larger than the term in Eq. (6) by a factor of $\ell/r_\mathrm{min} \gg 1$. Thus, if you choose one value of ξ over your entire surface, then one portion of the membrane will experience a drag that is either significantly too small or too large.

3. Remeshing procedure

Overall, I feel your remeshing idea is clever, and I would like to (1) better understand the details of the method and (2) see data that verifies the numerical implementation is working as expected. As I understand it, the remeshing prevents highly sheared or distorted meshes by introducing a purely mathematical step in which nodes move around, though the overall surface shape (and the fields on the surface) does not change. To carry this out, you introduce a free energy $\mathcal{F}$ of the mesh, and use a Lagrange multiplier field to enforce the constraint $\boldsymbol{v} \cdot \boldsymbol{n} = 0$. This seems quite similar to the implementation in Sec. 5.2 of Ref. [47], which in this case would yield

$$\frac{\partial \mathcal{F}}{\partial \boldsymbol{X}_a} + \int_\mathcal{S} p \boldsymbol{n} B_a \, \mathrm{d}S = \boldsymbol{0} \tag{9}$$

and

$$\int_\mathcal{S} \left(\boldsymbol{X}^{(n+1)} - \boldsymbol{X}^{(n)} \right) \cdot \boldsymbol{n} B_a \, \mathrm{d}S = 0 \,. \tag{10}$$

Equation (10) expresses the constraint in terms of $\boldsymbol{X}$ rather than $\boldsymbol{v} = \dot{\boldsymbol{X}}$, such that the virtual work associated with the Lagrange multiplier can be written as

$$\delta \left(\int_\mathcal{S} \left(\boldsymbol{X}^{(n+1)} - \boldsymbol{X}^{(n)} \right) \cdot \boldsymbol{n} \, p \, \mathrm{d}S \right) . \tag{11}$$

With this understanding, I have three clarifying questions:

- Why does v^α appear in Eq. (30)? It is dimensionally inconsistent with the pressure term, and seems out of place.
- The weak form of Eqs. (30) and (31) differ by a factor of time. Is this factor of "fictitious time," over which the mesh relaxes, absorbed into the Lagrange multiplier? You could verify this by determining the Lagrange multiplier for different fictitious time step values.
- Since $\mathcal{F}$ only contains elastic information, there is no timescale in the mesh relaxation. Do you introduce an artificial viscosity such that the mesh can slowly evolve, over the fictitious time?

In addition to these clarifications, you do not provide any information regarding the errors associated with the remeshing procedure. Given that in-plane flows (and thus mesh distortions) are a key component of your numerical implementation, I find this unacceptable. Could you please provide data on how errors in the (1) total surface area, (2) total volume, and (3) surface position scale with mesh refinement, and refinement of the fictitious time step? Furthermore, you do not discuss how you determine the velocity field on the relaxed mesh, and guarantee that it is the same velocity field as that on the initial, distorted mesh. I would also like to see how the error in the velocity field scales with mesh size and time step.

Minor Comments

1. On page 5, you say that with Eqs. (2)–(7), you have a complete set of equations to determine the velocity field $\boldsymbol{v}$. However, the kinematic relationship between $\boldsymbol{v}$ and $\boldsymbol{X}$ is required to close the problem.
2. When discussing irreversible thermodynamics at the bottom of page 5, and concentration fields involving binding and unbinding reaction rates on page 19, *Sahu et al.* Phys. Rev. E **96** (2017) should be cited.
3. You claim below Eq. (15) that your stiffness matrix is symmetric, however your equations are highly nonlinear and this statement goes against my intuition. In particular, I would imagine $\partial \boldsymbol{F}_a / \partial \boldsymbol{X}_b^{(n+1)} \neq \partial \boldsymbol{F}_b / \partial \boldsymbol{X}_a^{(n+1)}$.
4. Is there a reason that you present Eq. (18) in the main text, but relegate the expression which only involves normal displacements into the SI [Eq. (53)]? As you mention in the text below Eq. (20), it is more intuitive to see that the interaction term only involves normal displacements, and is unaffected by in-plane flows of the material that do not change its overall shape.
5. In your motivation for the free energy between two surfaces, wouldn't microscopic linkers only exert forces on cells that are fairly close together? It would be helpful to mention this at the outset, as this also explains why your interaction potential is short-range.
6. You do not mention needing to remove rigid body modes in your finite element scheme. Is this due to the external friction term $-\xi \boldsymbol{v}$? And consequently, if one wanted to set $\xi = 0$, could your numerical scheme be easily augmented to remove such rigid rotations and translations?

7. I am confusing by your single-cell result in Sec. 3.1. In your analytical solution, γ^{Aa}, ϕ^{Aa}, and thus v_n^{Aa} do not change in time. However, a nonzero v_n requires the surface to continually deform, and a steady state will never be reached. When (at what time in your simulation) are you calculating your error? In your simulation, do you eventually see a steady state where the shape stops evolving?

8. The convergence presented in Fig. 4F is not appropriate for demonstrating the validity of your finite element implementation. Please show appropriate convergence of the doublet position (in the same manner as the velocity in Sec. 3.1), at a fixed time, for fixed values of $\tilde{D}$ and $\tilde{\kappa}$.

9. There is a typo in Eq. (4) of the SI: the last term should evolve $\boldsymbol{e}_k$, not $\boldsymbol{e}^k$.
