# Supplementary material for: Interacting active surfaces: A model for three-dimensional cell aggregates
Source: PLoS Comput Biol. 2022 Dec 16;18(12):e1010762. doi: 10.1371/journal.pcbi.1010762 (PMC9803321; doi:10.1371/journal.pcbi.1010762)
Supplement: Attachment — Submitted filename: Response_reviewers.pdf [file pcbi.1010762.s007.pdf]

Reviewer 1

Summary

The study by Torres-Sánchez et al. describes a numerical method to simulate the mechanics of a collection of closed cells. The boundary of each cell is described as a two-dimensional surface, which represents the cell membrane and its attached acto-myosin cortex. The continuum equations governing the evolution of such a two-dimensional surface are taken from prior investigations by the authors (Ref. [52]) and are discretized with a Lagrangian finite element scheme that uses Loop subdivision surfaces to maintain the necessary surface continuity requirements. The numerical method captures in-plane flows and out-of-plane elasticity of each cell, as well as short-range intercellular adhesive forces between cells. The code for this method, written in C++, has already been openly released.

Given the variety of physical phenomena incoroporated into this numerical framework, and the public release of the numerical implementation, I believe this manuscript and its accompanying code are of timely interest to scientists in the field of cellular mechanics.

We thank the reviewer for his/her positive comments about our manuscript and his helpful comments.

Thus, I favor publication in PLOS Computational Biology if the authors can provide a satisfactory response to my three major concerns. One is related to the absence of a mass balance in the governing equations, which I fear may render the numerical implementation invalid for the types of phenomena the authors seek to describe. My second concern is regarding the possibility of lubrication layers to develop in the thin regions between cells. Finally, I would like to see numerical benchmarking for the remeshing procedure.

Major Comments

1. Mass balance and surface tension

I have a major concern regarding the “active tension” contribution to the in-plane stresses, expressed as $t^{ij}_a = \gamma g_{ij}$. When γ is prescribed to be a uniform constant, $\nabla_i t^{ij}_a = \gamma \nabla_i g^{ij} = 0$ and your active tension does not drive in-plane flows (it only contributes to an effective Laplace pressure in the out-of-plane equation). If γ is prescribed and non-uniform, you provide another forcing term $f^i_a = \nabla_i \gamma$ into the in-plane equations, which drives a flow of material. In both cases, however, you do not include a mass balance in your dynamical description—and so there is no coupling between areal dilations/contractions and the dynamics of the material. I find this extremely problematic: it may render all of your numerical results invalid, as I detail below.

Let us first consider the case where there are no active tensions. In this case, one could model the energetics of area changes as

$$W_{compress} = \int_S dS\{k_c J(J - 1)^2\}, \quad (1)$$

where kc is a compression modulus and $J = \rho0/\rho$ is the Jacobian determinant of the transformation—see, for example, Eqs. (153), (154), and (159) of Sahu et al. Phys. Rev. E 96 (2017). In this case, the contribution to the in-plane stresses is given by

$t\hat{}ij\_c = 2k\_c(J - 1)g\_ij$, (2)

which leads to an equation of state for the surface tension—written as

$\gamma\_c(\rho) = 2k\_c(J - 1)$. (3)

The resultant in-plane force in the linear momentum balance is given by

$\nabla it\hat{}ij\_c = -2k\_cJ\rho\nabla j\rho$. (4)

Such a term is missing from your analysis, and is likely to be important in many of the phenomena you seek to describe. In cases where you prescribe γ to be constant, the surface tension gradients in Eq. (4) would drive a lipid flow that your numerical method currently does not capture. On the other hand, if you prescribe γ to be spatially varying, then surface tension gradients could in fact balance the prescribed $\nabla\_i\gamma$, and your system could perhaps reach a steady state in which lipids are stationary—though in your current implementation, there must always be an in-plane flow of lipids, even at long times. Without a mass balance in your dynamical description of the system, it is hard to know if your numerical results even qualitatively describe biological situations, and thus your numerical method is incomplete.

We thank the reviewer for his/her comment. We agree that this point needs clarification in our manuscript.

Importantly, the surface flow we are describing corresponds to flows of the cell actomyosin cortex, which are thought to dominate cell surface tension compared to stresses in the lipid membrane. By fixing a uniform density, we assume that turnover of the components of the actomyosin cortex is much faster than the typical time-scale of cortex density change by flow and deformation. We have now added a sentence to explicitly mention this point (last sentence before section 2.1.3). Briefly, if the chemical rates of actin and myosin binding and unbinding at the cortex are fast enough, density perturbations caused for instance by cortical flows are suppressed and the density can be treated as homogeneous. We note that such an approximation has been extensively used previously in studies of cell mechanics (Refs. 31, 33-38).

I have one additional comment regarding your active tension term. If we think of γ as an effective energy per area, then to me it makes physical sense to think of γ as the product of an energy per mass ξ and the mass per area ρ. Then a “constant” active tension would be expressed as ξ = const., such that the active stresses would vary as material is compressed in-plane. Indeed, your own earlier work (Ref. [38], §3.3, §5.3) argues that the active surface tension should be expressed as

$\gamma = \rho\, \xi(\rho)$, (5)

and then proposes forms for $\xi(\rho)$. Even if ξ were independent of ρ, there is still some effect of active forces on the in-plane flow of materials. If you insist on prescribing γ with no mass balance, I fear that your numerical method would fail to reproduce the well-known instabilities in cylindrical membranes that have been observed in many experiments, including in live neurons with an intact acto-myosin cortex:

• Pullarkat et al. Phys. Rev. Lett. 96 (2006).

• Datar et al. Biophys. J. 117 (2019).

In such systems, a global disturbance to the system leads to pearling, which is theoretically known to arise when $\gamma > 3\kappa/(2R2)$—see, for example, Narsimhan et al. J. Fluid Mech. 777 (2015), which includes the effects of both membrane and fluid viscosity in the Appendix. A local perturbation, on the other hand, leads to outwardly propagating fronts, as discussed in Tchoufag et al. Phys. Rev. Lett. 128 (2022). I believe your current numerical implementation cannot describe a cylindrical cell which, when subject to different infinitesimal perturbations, evolves in these two different ways.

We thank the referee for these interesting comments. Here we are not assuming that the active tension does not depend on the cortical density, but we are taking the limit where the cortical density can be treated as uniform due to turn-over, as detailed above and now more explicitly mentioned in the manuscript.

We note that the Rayleigh-Plateau pearling instability can arise in a cylindrical system with uniform and constant surface tension, so strictly speaking our simulation would still capture this instability. However as the referee points out, this approximation might break down in regimes where the actin cortex is unstable, leading to cortical inhomogeneities and associated cell shape perturbations. In that case, the density of the actin and myosin at the cell surface have to be taken into account. In the discussion, we propose an extension of the model accounting for a concentration field, such as cortical density or myosin concentration, which can be easily introduced in our framework following the ideas in e.g. Ref. 38 (now Ref. 41). We are keen to consider this generalisation of our model in future work.

2. Lubrication layers between cells

To account for the effects of the internal and external medium surrounding the cell, you apply a body force $f = P\, n - \xi v$ to the cell surface. Here, P is meant to represent the pressure difference across the surface, and is approximated by the Lagrange multiplier enforcing the global volume constraint in Eq. (14). In this case, the internal pressure is assumed constant, and is measured relative to the external pressure—a fixed, reference value. Moreover, if the surface is surrounded by fluid, then the parameter ξ captures the drag due to shear forces, for which

$\xi \sim \mu_f\, l$, (6)

where μ_f is the fluid shear viscosity and l is a characteristic length scale (the effective radius you define, for example). While these approximations seem reasonable for a single cell, I worry about the lubrication effects that arise within the thin layer of fluid separating two cells, especially given that one of the key advancements of your method is the ability to simulate many adjacent cells. Let us consider two cells separated by a thin fluid region of width rmin and length l, with rmin ≪ l. If we denote the characteristic speed of fluid normal to the cell surface as vc and that parallel to the surface as uc, then it is well-known from lubrication theory that

uc/l = v_c r_min and ∆P_f = μf uc l r^2_min, (7)

where ∆P_f is the pressure drop of the fluid along the length l of thin region [L. Gary Leal. Advanced Transport Phenomena, Chapter 5. Cambridge University Press (2007)]. Lubrication pressures can be large, and could dominate the internal pressure P you determine from your volume constraint— yet this effect is missing from your implementation. Moreover, the shear force on the cell by the lubrication fluid now scales as μf uc/rmin, for which the corresponding drag coefficient would be

ξ ∼ μf r_min, (8)

which is larger than the term in Eq. (6) by a factor of l/rmin ≫ 1. Thus, if you choose one value of ξ over your entire surface, then one portion of the membrane will experience a drag that is either significantly too small or too large.

We thank the referee for this excellent and interesting comment. We agree that introducing the physics of lubrication layers between cells would be interesting in future work. At this stage, we have chosen to run simulations in a specific regime, where (i) dissipation is dominated by the viscosity of the cortex, (ii) as pointed out by the referee, a small external friction coefficient is introduced; this friction coefficient is small enough to have mostly the effect of damping translation and rotation modes, and does not play a significant role in setting the dynamics of the system. This is mentioned in section 3.2: “In the following we set $\tilde{\xi} = 10^{-3}$, so that the effect of friction is small”.

We also note that the surface is not only subjected to the cell pressure and external friction forces, but also to interaction forces that represent the effect of cell-cell linkers. At the moment, we only consider static contribution from linkers. In the future, we would like to introduce friction forces arising from linker redistribution as well, which might dominate lubrication forces mentioned by the referee. However, at this stage we think that this is outside the scope of the manuscript.

3. Remeshing procedure

Overall, I feel your remeshing idea is clever, and I would like to (1) better understand the details of the method and (2) see data that verifies the numerical implementation is working as expected. As I understand it, the remeshing prevents highly sheared or distorted meshes by introducing a purely mathematical step in which nodes move around, though the overall surface shape (and the fields on the surface) does not change. To carry this out, you introduce a free energy F of the mesh, and use a Lagrange multiplier field to enforce the constraint v · n = 0. This seems quite similar to the implementation in Sec. 5.2 of Ref. [47], which in this case would yield

$\partial F/\partial X_a + \int_S p_n B_a dS = 0$ (9)

and

$\int_S (X(n+1) - X(n)) \cdot n B_a dS = 0$. (10)

Equation (10) expresses the constraint in terms of X rather than $v = \dot{X}$, such that the virtual work associated with the Lagrange multiplier can be written as

$\delta(\int_S (X(n+1) - X(n)) \cdot n p dS)$. (11)

With this understanding, I have three clarifying questions:

• Why does v_α appear in Eq. (30)? It is dimensionally inconsistent with the pressure term, and seems out of place.

• The weak form of Eqs. (30) and (31) differ by a factor of time. Is this factor of "fictitious time," over which the mesh relaxes, absorbed into the Lagrange multiplier? You could verify this by determining the Lagrange multiplier for different fictitious time step values.

• Since F only contains elastic information, there is no timescale in the mesh relaxation. Do you introduce an artificial viscosity such that the mesh can slowly evolve, over the fictitious time?

We thank the reviewer for his/her comment. The physical picture is that the mesh relaxes the energy F following a gradient flow with a friction coefficient \xi_m. Therefore, the friction term proportional to v^{\alpha} in Eq. (30) is necessary to provide with a dynamics of mesh relaxation, ensuring that the mesh slowly evolves, as mentioned by the referee (although dissipation is ensured by an external friction, not by a mesh viscosity). Eqs. (30) and (31) are solved simultaneously to find the velocity field, with Eq. (31) ensuring that the normal part of the mesh flow vanishes in the continuum limit of infinitely small mesh size. The choice of the friction coefficient dimension is arbitrary given that the unit of time is irrelevant to this step which is occurring in fictitious time. We have rewritten this part to avoid confusion, explicitly introducing the friction coefficient in fictitious time \xi_m in Eq. 30 and providing with more details in the text.

In addition to these clarifications, you do not provide any information regarding the errors associated with the remeshing procedure. Given that in-plane flows (and thus mesh distortions) are a key component of your numerical implementation, I find this unacceptable. Could you please provide data on how errors in the (1) total surface area, (2) total volume, and (3) surface position scale with mesh refinement, and refinement of the fictitious time step? Furthermore, you do not discuss how you determine the velocity field on the relaxed mesh, and guarantee that it is the same velocity field as that on the initial, distorted mesh. I would also like to see how the error in the velocity field scales with mesh size and time step.

We agree with the referee that this point needed clarification in the manuscript.

- We have now added a new section to the supplementary text (S1 Appendix 7) clarifying how we handle error control in our reparameterization method. Briefly, we use an adaptive time-step that is controlled by a tolerance with respect to the error in the normal motion of the mesh.
- To test for error associated to the reparameterization, we have evaluated the distance between the shapes of the initial and relaxed meshes for different mesh size and different values of the tolerance. The result is shown in a supplementary video (S2). We find that the error is below 5.10^{-3} in unit of the radius of a spherical cell.
- Regarding the behaviour of the velocity field during mesh reparameterization: we actually do not need to calculate the velocity field on the relaxed mesh. Indeed, the velocity field calculated a time t has already been used to evolve the shape when we perform the mesh reparameterization step. In addition, because we work at low Reynolds number, the velocity field is entirely calculated at each time point, such that the velocity field at time t is not needed to solve for the velocity field at time t+dt.

Minor Comments

1. On page 5, you say that with Eqs. (2)–(7), you have a complete set of equations to determine the velocity field v. However, the kinematic relationship between v and X is required to close the problem.

We agree with the reviewer. We have now explicitly mentioned that we assume that X(s1,s2) is a Lagrangian parametrisation following material particles, and that v=\partial_t X.

2. When discussing irreversible thermodynamics at the bottom of page 5, and concentration fields involving binding and unbinding reaction rates on page 19, Sahu et al. Phys. Rev. E 96 (2017) should be cited.

We have added a citation to this reference.

3. You claim below Eq. (15) that your stiffness matrix is symmetric, however your equations are highly nonlinear and this statement goes against my intuition. In particular, I would imagine

∂F_a/∂X^(n+1)_b ≠ ∂F_b/∂X^(n+1)_a .

That the stiffness matrix is symmetric can be directly checked by inspection of the expressions in S1 (71-77) and (83,84). We now reference these equations in the main text.

4. Is there a reason that you present Eq. (18) in the main text, but relegate the expression which only involves normal displacements into the SI [Eq. (53)]? As you mention in the text below Eq. (20), it is more intuitive to see that the interaction term only involves normal displacements, and is unaffected by in-plane flows of the material that do not change its overall shape.

We believe that this result being relatively technical, it is better done in details in the Supplementary to avoid overwhelming the reader of the main text.

5. In your motivation for the free energy between two surfaces, wouldn't microscopic linkers only exert forces on cells that are fairly close together? It would be helpful to mention this at the outset, as this also explains why your interaction potential is short-range.

We have added an additional schematic to clarify our physical picture for cell-cell interaction from the outset.

6. You do not mention needing to remove rigid body modes in your finite element scheme. Is this due to the external friction term $-\xi v$? And consequently, if one wanted to set $\xi = 0$, could your numerical scheme be easily augmented to remove such rigid rotations and translations?

This is indeed due to the external friction term. In principle, a set of Lagrangian multipliers could indeed be added to constraint global translation and rotations. However, because this would couple the equations for different cells which would significantly slow down the numerical solving, it is more efficient computationally to introduce an external friction term.

7. I am confusing by your single-cell result in Sec. 3.1. In your analytical solution, γAa, φAa, and thus vAan do not change in time. However, a nonzero vn requires the surface to continually deform, and a steady state will never be reached. When (at what time in your simulation) are you calculating your error? In your simulation, do you eventually see a steady state where the shape stops evolving?

The referee is correct, but, in our analysis, we only consider the flow calculated at the first time point where the sphere has not yet deformed. This is required for the calculation in spherical harmonics to be exact, and is therefore well suited to comparison between analytics and simulation results. We have now edited the main text to clarify this point.

8. The convergence presented in Fig. 4F is not appropriate for demonstrating the validity of your finite element implementation. Please show appropriate convergence of the doublet position (in the same manner as the velocity in Sec. 3.1), at a fixed time, for fixed values of D˜ and ˜κ.

Following the referee's suggestion, we have now added a convergence plot (now in Fig. 5F) where we compare the velocity fields of the doublet in the initial configuration, as was done for single cells in Section 3.1. This comparison allows to test convergence in a convenient manner, as the initial shape is spherical which allows for an easy mapping between meshes of different sizes. The plot showing convergence of pressure is now shown as a supplementary panel in Fig. S3.

9. There is a typo in Eq. (4) of the SI: the last term should evolve e_k, not e^k.

We thank the reviewer for his/her thorough examination of the equations, this is now corrected.

Reviewer 2

The interest in modeling biological surfaces is not recent, but most studies mainly focused on passive membranes. More recently, several papers extended the study of fluid surfaces to active surfaces, which is a natural and necessary step to making better models with the hope of quantitatively confronting theory with biological experiments. This article provides significant advances for the simulation of three-dimensional biological systems that will undoubtedly positively impact the field. The authors put forward a numerical approach capable of handling multicellular aggregates with the dynamical evolution of surface fields. There is a good balance in the article between theory (where there is nothing particularly new, but this is not the article's focus), numerics, and results. The cell mechanics is dictated here by the actomyosin cortex at a long (viscous) timescale, and cell-cell adhesion is modeled using the phenomenological Morse potential. They present biologically relevant applications of the numerical scheme. The paper is well written and would appeal to what I consider typical readers of PLOS. After some minor revisions, I would be happy to recommend it to the editors of PLOS for publication.

We thank the referee for his positive evaluation of our work and for his insightful comments.

Introduction:
In the introduction, the reader might benefit from a brief discussion on the physical/biological motivation on why only the cortex is necessary for describing the cell dynamics and why the membrane plays no role.

We agree and have added a sentence in the introduction to clarify this point.

The authors say on pg. 2, "In this framework, topological transitions appear as a natural output of the remodeling of cell-cell interactions and are not treated explicitly." However, it is not clear how the topological transitions will happen naturally. This is particularly relevant because Sec 3.4 deals with cell divisions. Still, the authors chose to cut the cell in a random plane instead of modeling the division dynamics (as in the ref. HB da Rocha, J Bleyer, H Turlier - Journal of the Mechanics and Physics of Solids, 2022, although I believe that here the authors did not treat topological transitions).

We agree and have replaced “topological transitions” by “neighbour exchange events”, which is the case we were thinking about when writing this sentence.

Materials and methods

Pg. 4:

I'm curious to know why the authors chose the virtual work principle to formulate what is clearly a fluid problem. Wouldn't the Onsager variational principle or virtual power principle be more relevant here? I believe that because they deal essentially with stokes flow, ultimately, there will be no problem.

This is an interesting point; in fact we have used the virtual work principle because it is an exact statement of force balance in any limit and for any rheology, provided that inertial forces are introduced as external forces. This is discussed in e.g. Salbreux et al, PRE, 2017.

Pg. 8:

The section on the forces arising from cell-cell interactions is presently a bit confusing. The authors start by stating a general function for the cell-cell interactions, then go on to motivate a particular form for the cell-cell interaction energy considering stretchable linkers connecting the cells with binding and unbinding dynamics. Then, the authors observe that such potential fails to capture repulsion and turns to a phenomenological potential, the Morse potential. I believe that this section could go direct to the point.

We agree with the referee and had the same hesitation in how to present our results. Our conclusion was that, even if in practice we use a phenomenological Morse potential, it is helpful to see that a clear connection can be made between the effective potential and the microscopic dynamics, and therefore that this is an important result. Notably, our calculation leading to Eq. (24) could be used in future work, if a more detailed knowledge of the potential phi becomes available. To avoid possible confusion pointed out by the referee, we have added a new schematic to clarify our physical picture and reorder the different parts of the section.

Pg. 11:

The authors devote a subsection to presenting an already established surface reparametrization method. Yet, the exposition is restricted to a single cell without discussing the reparametrization in the numerical experiments. Thus, I would suggest the authors either shorten the exposition to a minimum or complete it with (1) information about the extension to cell aggregates and (2) error assessment in the numerical examples to give the reader an idea of the accuracy of the method. For instance, are there any difficulties when parametrizing cell aggregates (e.g., possible self-intersections)? What is the reparametrization error in terms of volume conservation?

To our knowledge, a reparametrisation method like ours is not present in the literature, even though there are clear similarities with the method by Klug and colleagues (refs. 63 and 64). We agree with the reviewer that the error assessment of the method was not appropriately presented nor was how this method is incorporated in the dynamics of an aggregate. We have now added a description and a supplementary video of a prototypic example of mesh relaxation following our method for different mesh sizes and different values for the tolerance in the error in normal motion. We have also explicitly mentioned that, as our method does not change cell shape (up to numerical errors now discussed in Supplementary (S1 appendix 7) that are very small) it can be applied independently to each cell of the aggregate without resulting into issues such as self-intersections.

Pg. 20:

In the discussion section, the authors report that the exact integration of the interaction potential has a high computational cost. I think the reader would also benefit from knowing the relative weight of this simulation phase, i.e., the percentage of time devoted to integrating the interaction potential with respect to the total simulation time.

Finally, the authors highlight in the abstract and the introduction their hybrid MPI-OpenMP parallelized C++ implementation. Yet, the reader is given very few details in the subsequent exposition on the parallelization, especially in the numerical experiments. I would suggest reporting at least minimal quantities to give the reader an idea of the parallel performance of the code. For instance, to inform about global problem sizes, max. local problem sizes, max. number of cores employed, total simulation times, and CPU specification for the largest numerical examples.

We thank the reviewer for this suggestion. We have now included a new section in the Supplementary Information (S1 appendix 11) describing code performance achieved in the second numerical example discussed in the manuscript.

Reviewer 3

This work presents a general computational model for the interaction of active fluidic surfaces. The manuscript is very well written, interesting and important. The presented examples are well chosen and clearly demonstrate the versatility of the proposed model. The work is suitable for publication provided the following comments are addressed:

We thank the referee for his positive evaluation of our work and his helpful comments.

1. Sec. 2.1: Usually the exact opposite notation is used: Greek indices for surface coordinates and Latin indices for 3D coordinates. The authors should motivate their choice if it is non-standard.

We would argue that there is no standard choice for Greek and Latin indices for 2D and 3D coordinates, for instance the following references from other authors use the same convention as us:

- Arroyo, M., & Desimone, A. (2009). Relaxation dynamics of fluid membranes. *Physical Review. E, Statistical, Nonlinear, and Soft Matter Physics*, *79*(3 Pt 1), 031915.
- Nestler, M., Nitschke, I., & Voigt, A. (2019). A finite element approach for vector- and tensor-valued surface PDEs. *Journal of Computational Physics*, *389*, 48–61.
- Capovilla, R., & Guven, J. (2002). Stresses in lipid membranes. *Journal of Physics A: Mathematical and General*, *35*(30), 6233–6247.
- Naji, A., Atzberger, P. J., & Brown, F. L. H. (2009). Hybrid elastic and discrete-particle approach to biomembrane dynamics with application to the mobility of curved integral membrane proteins. *Physical Review Letters*, *102*(13), 1–4.

As we have used the same choice in some of our earlier work (e.g. Salbreux&al, PRE, 2017), we would like to stick to our current notation.

2. Sec. 2.1: g_ij, C_ij, etc. are not tensors (even if many authors erroneously call them so). They are just components, possibly of tensors. The full tensor includes the basis vectors, which makes a big difference, esp. for curvilinear coordinates.

We agree that strictly speaking, the referee is correct; however, as he/she points out, this slight abuse of notation allows to shorten notations and is used very frequently in the literature, see e.g. 3 examples from different fields:

Capovilla, R., Guven, J., & Santiago, J. (2003). Deformations of the geometry of lipid vesicles. *Journal of Physics A: Mathematical and General*, *36*(23), 6281.
Lovelock, D. (1971). The Einstein tensor and its generalizations. *Journal of Mathematical Physics*, *12*(3), 498-501.
Kruse, K., Joanny, J. F., Jülicher, F., Prost, J., & Sekimoto, K. (2005). Generic theory of active polar gels: a paradigm for cytoskeletal dynamics. *The European Physical Journal E*, *16*(1), 5-16.

Here as well, we would like to keep a notation consistent with our earlier work to help the interested reader (e.g. Salbreux&al, PRE, 2017).

3. Following Eq. (7): The authors should note here that the scope of constitutive laws for fluidic membranes as it stems from irreversible thermodynamics has also been discussed in Sahu et al. (2017), “Irreversible thermodynamics of curved lipid membranes”, Phys. Rev. E, 96:042409

We thank the referee for pointing out this work to us and have added this reference to the manuscript.

4. Sec. 3.1: It is not clear to me, whether the surface shape changes in this example. There is an out-of-plane surface velocity but then the analytical solution seems to be based on a spherical shape.

The referee is correct that there is an out-of-plane surface velocity. However, in our analysis, we only consider the flow (including a normal component) calculated at the first time point, when the sphere has not yet deformed. Indeed we have to restrict ourselves to a spherical shape in order for the analytical calculation in spherical harmonics to be exact. This choice is therefore well suited for comparison between analytics and simulation results. We have now edited the main text to clarify this point (section 3.1).

5. It is not clear to me to what degree the surface flow has an influence on the presented results in Sec. 3.2. & 3.3. (and also 3.4). Did the authors vary the relevant parameters to examine? It would be interesting to see this.

We agree with the referee that this is an interesting question which we also asked ourselves; notably to what extent the cell surface viscosity can influence our results.

In section 3.2, we show the dynamics of contact growth in Fig. 5A as a function of time normalized by tau. The only parameter affecting the dynamics is this parameter tau, which depends on the cell surface viscosity and is used for time normalization.

Therefore, here changing the cell surface viscosity leads to a simple rescaling of the dynamics.

In section 3.3, we consider a steady-state situation where the surface flow does not influence the result. This is mentioned in section 3.3 “we let the system relax to its equilibrium state”. We also expect here that the relaxation to steady-state will be controlled by the parameter tau.

In section 3.4, we introduce another timescale t_D corresponding to the time between cell divisions. Therefore, there we explored the effect of varying the ratio tau/t_D. As can be seen in Fig. 7D, this leads to a significant change in the shape of the simulated growing cell aggregate.

6. At some point the authors mention that the active surface tension is considered a given constant, but in principle could depend on some concentration and vary locally. Is this the case in all four examples? The authors should clarify.

We agree with the referee. We have now clarified that we consider the limit where the cortical density, and therefore the active surface tension, is homogeneous (see new sentence just before section 2.1.3). We think that relaxing this assumption is a great avenue for future work, and we now mention this point explicitly in the discussion.
