# Supplementary material for: Interacting active surfaces: A model for three-dimensional cell aggregates
Source: PLoS Comput Biol. 2022 Dec 16;18(12):e1010762. doi: 10.1371/journal.pcbi.1010762 (PMC9803321; doi:10.1371/journal.pcbi.1010762)
Supplement: Attachment — Submitted filename: Reply.pdf [file pcbi.1010762.s008.pdf]

We thank the referee for his positive evaluation of our revision and for his interesting comments. We provide a detail response to his comments below.

I thank the authors for their careful responses to my comments, as well as the comments of the other reviewers. I am satisfied with the response to my minor concerns, as well as two of my three major concerns. However, I remain worried about your treatment of the active tension, in which γ is either a prescribed constant or a prescribed field (which does not vary in time). I now understand that you seek to describe a cell in the limit where cytoskeletal elements are constantly and rapidly turning over, such that local inhomogeneities are quickly removed and can be neglected on the natural timescale of membrane deformations/flows. In my mind, I picture a reservoir of cytoskeletal elements in the cell cytoplasm to allow such a situation. However, this brings up additional questions:

The referee is raising 4 points. Briefly, regarding point 4, we show below numerically and analytically that the Plateau-Rayleigh does occur in our framework. Regarding points 1-3, the referee is raising interesting questions to which we reply below. We note that these comments are relevant not only to our work, but more generally to the large body of literature which treats the cell surface as an active fluid (please see for instance Refs. 31, 33-38 in our manuscript). Our study makes modeling choices consistent with this litterature. It would be interesting to go beyond the state of the art in the hydrodynamic description of the actomyosin cortex, but we believe that this would be beyond the scope of our study.

1. It seems such a composite system (membrane + cortex) is no longer described by the Helfrich free energy, which is appropriate for a two-dimensional system with in-plane fluidity. Given your statement that cortical stresses dominate membrane ones, wouldn't an energy density for a material with in-plane elasticity (and not fluidity) and out-of-plane bending be more appropriate? Your edits to the introduction say that the composite system is in-plane fluid on the timescale of tens of seconds, but many cellular phenomena of interest happen on shorter timescales. Wouldn't your choice of free energy then imply you cannot resolve any of those?

As is now clarified in the introduction, we describe the system on time-scales for which in-plane elastic stresses are relaxed, and therefore we do not include in-plane elasticity in the model. A more refined description could capture viscoelastic stresses arising in the cortex (rather than purely viscous) with a Maxwell model, in which elastic stresses relax on a characteristic Maxwell time. In its present form, our model can not capture cortical flows occuring on timescales shorter than the Maxwell time, and we can only describe cellular phenomena which occur on time scales larger than tens of seconds. We argue that this is however relevant to many cellular processes, for instance cell growth, cell division and cell migration which occurs on timescales of tens of minutes to hours. We note that this approach is consistent with state-of-the-art litterature (Ref 31, 33-38).

2. Generally, a bending deformation costs energy because one side of the sheet is stretched while the other is compressed, both of which are energetically unfavorable. However, if the turnover of cytoskeletal elements is so rapid, wouldn't this bending energy no longer exist, since there is no way for stress to be built up? Rather, the actomyosin cortex

would always remain in a stress-free configuration. It seems the cortex turnover would have to in fact be slow relative to shape changes in order for bending forces to arise.

This is an interesting point. We argue that there is in fact in general no contradiction in a material being elastic with respect to bending deformations, and viscous with respect to in-plane deformations. The picture proposed by the referee applies for a thin layer of homogeneous material. However, the actomyosin cortex is a complex material, and is not homogeneous. We are not aware of studies which would specifically study the microscopic origin of the actomyosin cortex bending rigidity. However we can speculate: actin filaments have a finite length and a bending rigidity, so that bending the actomyosin cortex would cost actin filament binding energy; even if actin filaments are also free to rearrange with each other in the plane of the cortex. Turnover does not change this picture, because newly added filaments still have to bend to conform to the surface shape.

3. Even under the assumption that a rapid turnover prevents any density changes of the cortex, and that the Helfrich energy remains appropriate, wouldn't the lipid membrane itself still undergo density variations as the composite system deforms? In this case, even if the density of the cortex is constant, there will still be surface tension gradients caused by the membrane. Moreover, since the gradient of the "cortex" surface tension is zero, it is these membrane surface tensions which will dominate in the in-plane equations. A mass balance would then be required in your formulation.

This is an interesting point. We operate under the assumption that stresses at the cell surface are dominated by the actomyosin cortex, and our approach is consistent within this picture. We believe that the interplay of membrane and actomyosin cortex that the referee is pointing to is a very interesting question, but still poorly understood. We can envision that several effects could play a role in limiting the role of membrane stresses: (i) the cortex and membrane can in principle flow relative to each other; therefore the lipid membrane does not necessarily undergo density variations, even if compressive or extensile flows exist in the cortex; (ii) even if the cortex and membrane are flowing together, the membrane could buckle and thus provide limited residual stresses; or processes of endocytosis and exocytosis, adding and removing lipids from the membrane, could locally restore the equilibrium membrane density. Here as well, more refined descriptions of the actomyosin cortex and lipid membrane would be interesting to develop, but are beyond the scope of our current work.

We respectfully disagree with the fact that the gradient of cortical surface tension vanishes because of force balance, implies that membrane surface tension dominate the in-plane equations. The requirement of vanishing gradient of cortex surface tension sets a balance between viscous stresses and active stresses, whose gradients have to be opposite to each other. This balance determines the velocity profile in the cortex. Further incorporating membrane stresses in this picture, small in magnitude compared to both viscous and active stresses, would only slightly perturb this balance and the cortex velocity profile.

4. I believe your response regarding the Rayleigh–Plateau instability is incorrect. It is true that pearling arises in a cylindrical fluid column with constant surface tension. However, here the driving mechanism is the development of pressure gradients in the fluid column in response to an initial perturbation. These pressure gradients are due to the

Young–Laplace equation (constant tension and curvature gradients lead to pressure gradients), and drive a viscous fluid flow (via the Stokes equation) which reinforces the initial disturbance. However, since your numerical method does not capture any pressure gradients in the surrounding medium, I believe it cannot describe such an instability. If I am correct, then the inability of your equations to describe such a well-known and commonly observed cellular phenomena would be very troubling, and would further confirm my belief that you need an explicit statement of local mass balance in your dynamics.

We stand by our statement regarding the Plateau-Rayleigh instability. We have directly verified that the Plateau-Rayleigh occurs in our simulation, please see figure below. In this simulation, we consider a periodic tube of initial radius $R = 1$ and length $L = 10$ and with $\eta = 1, \gamma = 1, \bar{\eta} = 0$ and small friction $\zeta = 10^{-2}$ to prevent rigid body motion; the cylinder develops a Plateau-Rayleigh instability.

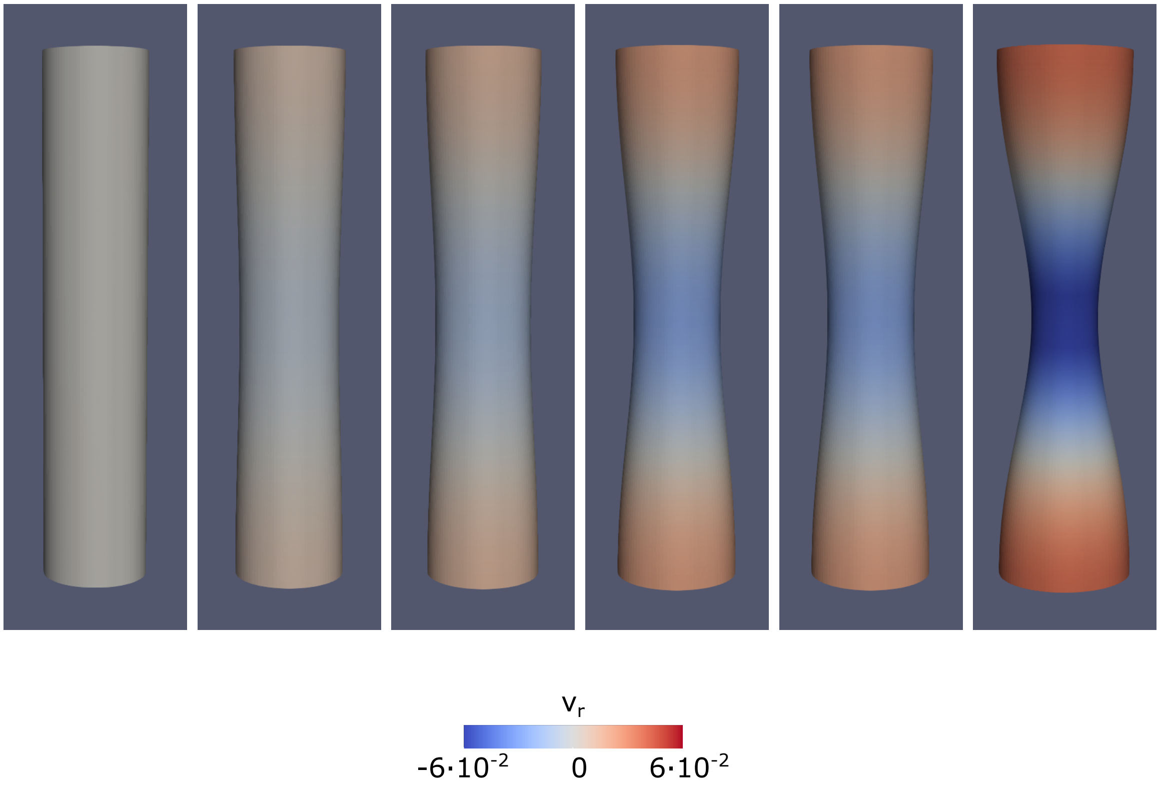

Figure 1. Sequence of snapshots of the onset of the Plateau-Rayleigh instability in a simulation of a periodic cylinder. The colormap indicates the radial velocity.

The viscous flow inside a fluid column that the referee is mentioning is a dissipative mechanism which controls the speed at which the instability arises, but has not effect on

the stability of the cylinder itself (and does not affect the stability boundary in a stability diagram). In other words, the driving mechanism for the instability is not coming from the fluid inside the cylinder, but from the fact that a sufficiently long cylindrical shape does not have minimal surface area, and the surface tension tends to minimize surface area. To make this point completely clear, below we calculate explicitly the stability of a cylinder under constant surface tension, when the pressure inside the surface is uniform, and flows are limited by the viscosity of the surface. As expected, we do find the standard Plateau-Rayleigh instability in this calculation.

We follow notations in Khoromskaia&Salbreux (https://arxiv.org/abs/2111.12820), which deals with axisymmetric surfaces, which are generally consistent with notations in the current manuscript. We consider a surface with tension tensor

$$t_{ij} = (\gamma + \bar{\eta} v_k^k) g_{ij} + \eta v_{ij} \tag{1}$$

with γ a surface tension, $\bar{\eta}$ and η are the bulk and shear viscosity of the surface, and v_{ij} the shear tensor. We consider an axisymmetric surface described by an arclength coordinate s going along the axis of symmetry, and the angle ϕ around the axis of axisymmetry. x denotes the distance of a point on the surface from the axis of symmetry. The curvature components are C_s^s, C_ϕ^ϕ. In that case, the tangential and normal force balance equations read:

$$\partial_s t_s^s = \frac{\cos\psi}{x}(t_\phi^\phi - t_s^s) \tag{2}$$

$$C_k^k t_s^s + C_\phi^\phi (t_\phi^\phi - t_s^s) = P \tag{3}$$

where P is the pressure inside the surface, assumed to be homogeneous. Here $\cos\psi = \partial_s x$. For a cylinder of radius R, $C_k^k = 1/R$, $\psi = \pi/2$ and a steady state solution exists for $\gamma/R = P$, as given by the normal force balance equation.

We now consider small deviations from this axisymmetric shape, with distance from the axis of symmetry $x = R + \delta r$. The normal velocity v_n satisfies $v_n = \frac{d\delta r}{dt}$. One then has

$$v_k^k = \partial_s v^s + \frac{v_n}{R} \tag{4}$$

$$v_s^s = \partial_s v^s \tag{5}$$

$$v_\phi^\phi = \frac{v_n}{R} \tag{6}$$

$$C_k^k = -\partial_s^2 \delta r - \frac{1}{R^2}\delta r \tag{7}$$

and the tangential and normal force balance equation now gives, writing $t_s^s = \gamma + \delta t_s^s$:

$$\partial_s \delta t_s^s = 0 \tag{8}$$

$$\gamma \left(-\partial_s^2 \delta r - \frac{1}{R^2}\delta r \right) + \frac{1}{R}\delta t_s^s + \frac{\eta}{R}\left(\frac{v_n}{R} - \partial_s v^s \right) = \delta P \tag{9}$$

or since $\delta t^s_s = (\bar{\eta} + \eta)\partial_s v^s + \bar{\eta}\frac{v_n}{R}$:

$$\frac{\eta}{R^2}\frac{2\bar{\eta} + \eta}{\bar{\eta} + \eta}\frac{d\delta r}{dt} = \gamma\left(\partial^2_s \delta r + \frac{1}{R^2}\delta r\right) + \delta P + \frac{1}{R}\frac{2\eta + \bar{\eta}}{\eta + \bar{\eta}}\delta t^s_s \tag{10}$$

with δt^s_s a constant. Performing a Fourier transform in space, the last two terms in the right-hand side only contribute to the mode 0 dynamics, describing uniform changes in radius of the cylinder. If the volume of the cylinder is conserved and the cylinder has a fixed length L, δP and δt^s_s adjust to suppress this mode of deformation. Other modes with Fourier wave vector k satisfy the dispersion relation

$$\frac{\eta}{\gamma}\frac{2\bar{\eta} + \eta}{\bar{\eta} + \eta}s = (1 - k^2R^2) \tag{11}$$

where one has also performed a Laplace transform in time with Laplace coordinate s. Here one recognizes the characteristic form of the Plateau-Rayleigh instability, with the dispersion relation proportional to $1 - k^2R^2$. This implies that only sufficiently long modes with $k < 1/R$ are unstable, the classical result from Plateau-Rayleigh.
